# Algae explosive growth mechanism enabling weather-like forecast of harmful algal blooms

**DOI:** 10.1038/s41598-018-28104-7

**Published:** 2018-07-02

**Authors:** Rongxiang Tian, Jianfang Chen, Xiangwei Sun, Dewang Li, Chongxuan Liu, Huanxin Weng

**Affiliations:** 1 Institute of Environment & Biogeochemistry, Zhejiang University, 310027 Hangzhou, China; 2 Key Laboratory of Marine Ecosystem and Biogeochemistry, Second Institute of Oceanography, SOA., Hangzhou, 310012 China; 3 School of Environmental Science and Engineering, Southern University of Science and Technology, Shenzhen, 518055 China

## Abstract

As a global problem in coastal environments, harmful algal blooms (HABs) have seriously affected the health of coastal ecosystems and regional economies. Here we report an aerosol-trigger mechanism for the occurrence of HABs based on long-term field data and laboratory experiments. The occurrence times of HABs and aerosol events had a significant correlation from 2005 to 2013 in the East China Sea, indicating that aerosol transport was probably an alternative trigger of HABs. HABs mostly occur in the transition time between winter and summer, during which northwest monsoon transport substantial aerosol (rich in phosphate, iron and other trace metals) to coastal waters, as revealed by chemical measurements, transmission electron microscope and electron microprober results. Such nutrients can stimulate algal growth in our incubation experiments, suggesting that such aerosol transport can be important nutrient sources for the East China Sea where phytoplankton growth is relatively phosphate limited. Air-borne nutrients are available for algal growth by rapid downward air flow, which additional results a clear weather condition, and thus adequate light intensity for algal growth. At last, the transition from northwest monsoon to warm southwest monsoon establishes favorable seawater temperature for algal blooms. Such weather-related aerosol-trigger mechanism suggests possibly forecast of HABs.

## Introduction

Harmful algal blooms (HABs) are rapid increase or accumulation in the population of algae. It colors the water, produces toxic^1,2^, and directly affects zooplankton^3^, fish^4,5^, and shellfish^6^ in coastal marine environment. Moreover, the falling and subsequent break down of biogenic particles may result in bottom hypoxia^7,8^ and subsurface acidification^9^ in marginal seas, which are intolerable for benthic life^8^. The frequently occurrence of HABs cost millions of dollars alone in China annually^10^. Thus, forecasting the severe HABs events based on its occurrence mechanisms is extremely important for policy-maker, fish man and aquaculture industry.

Field observations and simulations have revealed that the occurrence of HABs is resulted by a complex interaction of meteorological and biogeochemical factors^11^. Atmospheric aerosol transport was reported to be an important regulator of HABs^12,13^. It carried substantial nutrients^12^ and trace metals^14^ into coastal waters, which are also influenced by riverine nutrient input^15,16^. Such nutrient transport is especially important if riverine nutrient is consumed rapidly by phytoplankton, resulting nutrient limitation^17,18^. It is unclear, how the meteorology induces biogeochemical response, and generates a favorable condition for HABs in coastal waters.

Coastal waters of East China Sea have more than thirty algae bloom events annually^19^, also its chemical compositions are influenced by aerosol transport from Loess Plateau^20–22^. Moreover, phytoplankton growth is reported to be limited by relatively low dissolved inorganic phosphate concentration which is depleted much quicker than nitrate and silicate^17,23^. Thus, coastal water of East China Sea is an ideal place to study the relationship between atmospheric transport and HABs. In this study, we present HABs and aerosol event frequencies in the East China Sea, chemical compositions of aerosol in coastal cities, and laboratory experiments regarding the algal growth in different biogeochemical and light environments.

## Results

### Harmful algal blooms and aerosol events in the East China Sea

**Figure 1:**
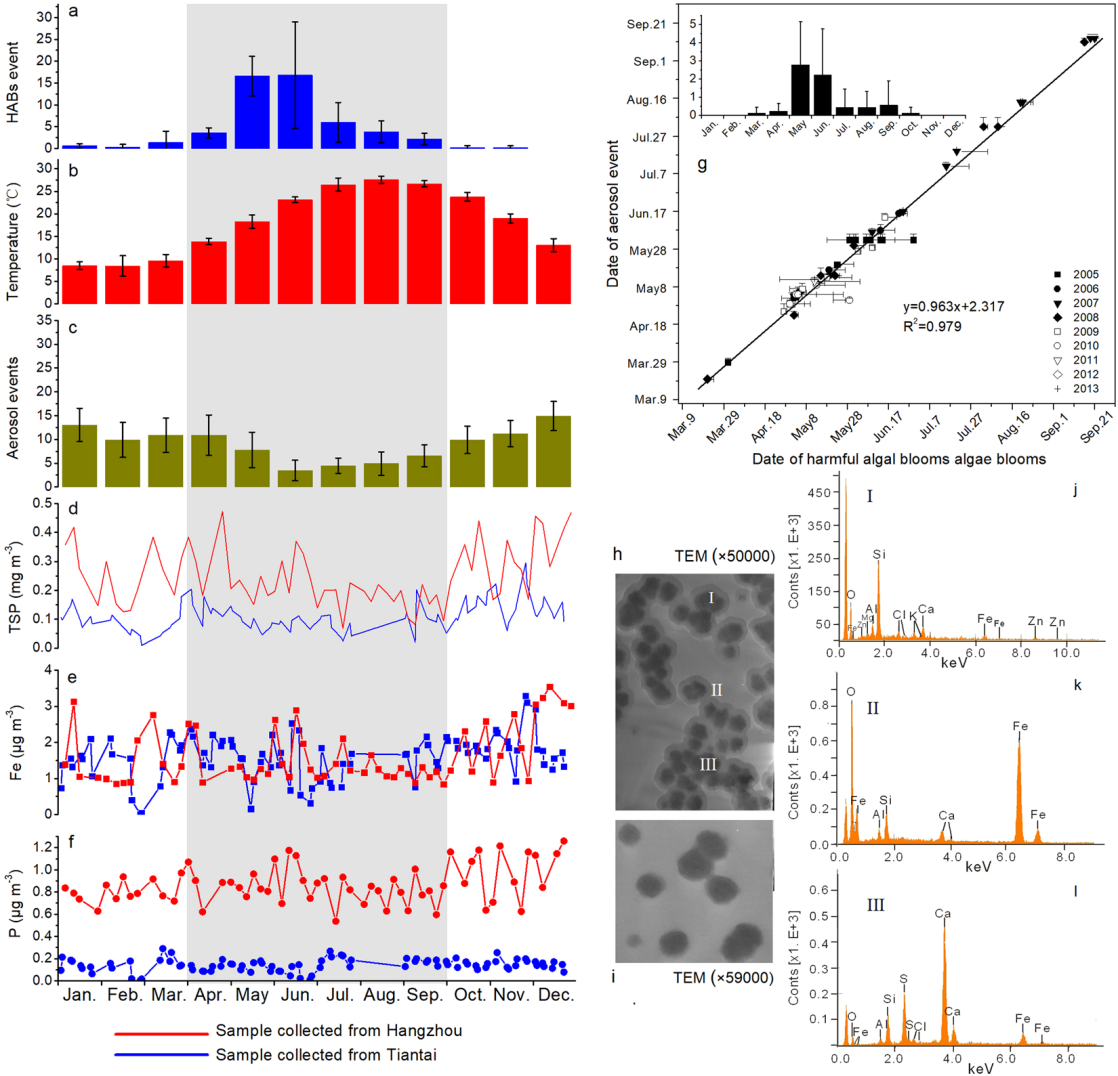
Monthly data in the East China Sea during 2005–2013 (Error bar represents one standard deviation) (**a**) HABs occurrence frequency; (**b**) mean sea surface temperature; (**c**) aerosol event frequency; (**d**) TSP concentration in atmosphere; (**e** and **f**) Fe and P concentrations of aerosol particles, respectively; (**g**) relationship between HABs occurrence and aerosol event; (**h**) TEM of aerosol particles (TEM images revealed that large grains are surrounded by colloidal materials. The mineralogical composition of the large grains is dominated by silicate (I, EMP result in **j**) and calcium-containing minerals (III, EMP result in **l**), while iron and other micronutrients are the major chemical composition in the colloidal parts (II, EMP result in **k**)); (**i**) TEM images of aerosol particles after contacting with sea water. In the left top of **g**, monthly variations of larger-scale HABs events (>300 km^2^) were also shown.

HABs primarily occurred from April to September in the East China Sea (Fig. 1a). From 2005 to 2013, algal species involved in the HABs events varied little, which were dominated by *Skeletonema costatum Cleve, Prorocentrum dentatum*, and *Karenia mikimotoi Hasen*^19^. The average surface seawater temperature ranged from 15 to 26 °C during the HABs months (Fig. 1b). Aqueous Fe concentrations in the East China Sea were within the range of 0.0006–0.010 μmol L^−1 ^^24,25^. In addition, although dissolved inorganic nitrate concentration in the front zone (HABs mostly occurred) was higher than 5 μmol L^−1^, phosphate concentration was only 0–0.20 μmol L^−1^ (Fig. S1). Such Fe and P may be not enough for sustaining explosive algae growth, especially for phosphate which may limit phytoplankton growth in the coastal East China Sea with limited phosphate concentration in the sea water^17^. In the coastal area of East China Sea, the molar ratio of dissolved inorganic nitrogen and dissolved inorganic phosphate (N:P ratio) is typically higher than 50. Consequently the dissolved inorganic phosphate will be exhausted faster than dissolved inorganic nitrogen if Redfield ratio of 16 is applied (The classical N:P ratio of marine plankton is 16:1)^16,17,26^.

The aerosol events occurred throughout year in the East China Sea (Fig. 1c). Remarkably, the occurrence of large-scale HABs (>300 km^2^) was temporally correlated with aerosol event (Fig. 1g), showing a significant correlation (r^2^ = 0.98, p < 0.01) between HABs and aerosol events with a lag time of a few days during the HABs months. There may exist a biogeochemical connection between HABs and aerosol transport in the East China Sea, as inferred from Hsu *et al*.^25^.

### Atmospheric iron and phosphate transport for algal blooms

To interrogate the potential causal relationship between HABs and aerosol events, atmospheric particles were collected on the route of aerosol migration from inland China to the East China Sea (suburban of Hangzhou and Tiantai cities). Total suspended particles (TSP) concentration varied significantly with time during HABs months (Fig. 1d). The collected particles were rich in iron (Fe), phosphate (P), and other micronutrients Co, Cr, Cu, Mn, and Zn (Figs 1e,f and S2). Previous study have indicated that trace metal concentrations during aerosol events are larger than that during non-aerosol events^22^. Furthermore, transmission electron microscopy (TEM) and electron microprobe (EMP) analysis of the aerosol particles revealed that Fe, P and other micronutrients primarily existed as coating or cementing materials that connected Si or carbonate mineral particles together (Fig. 1h,j,k,l). When these particles contacted with sea water, the coating and cementing materials dissolved, making nutrient elements exist in aqueous phase (Fig. 1i). Fe transported by aerosol in the China coastal waters was also confirmed by TEM results in the latest research^27^.

Vertical air-velocity analysis indicated that downward air flows prevailed during HABs events in the East China Sea (Fig. S3). Such downward flow suggested nutrients in the aerosol particles would probably precipitate and be available for algal. The atmospheric Fe flux to East China Sea could be 0.01–0.1 Mt a^−1^, larger than the riverine Fe input (~0.02 Mt a^−1^)^25^. In addition, Kim *et al*.^28^ showed that atmospheric precipitation increased N concentration in the East China Sea and northwestern Pacific Ocean. Utilizing Climate Nested Air Quality Prediction Modeling System (NAQPMS)^29^, the average concentration of N carried by the downdraft air reached 3296.03 nmol m^−2^ two days before and first four days of the HABs event (May 20 to 27, 2006). Although riverine material (Fe, P, N and Si) were important nutrient sources for coastal waters^30,31^, most of them may be consumed completely during long–distance transport due to intense phytoplankton consumption in the Changjiang River plume^16,32^ and nutrient deposition with particles^33^. Consequently, riverine nutrients may not be able to meet the continuous nutrient requirement of phytoplankton bloom, particularly for those large-scale HAB events that expanded several thousand square kilometers. On the other hand, there is no transport limitation for the air-borne Fe and P.

**Figure 2:**
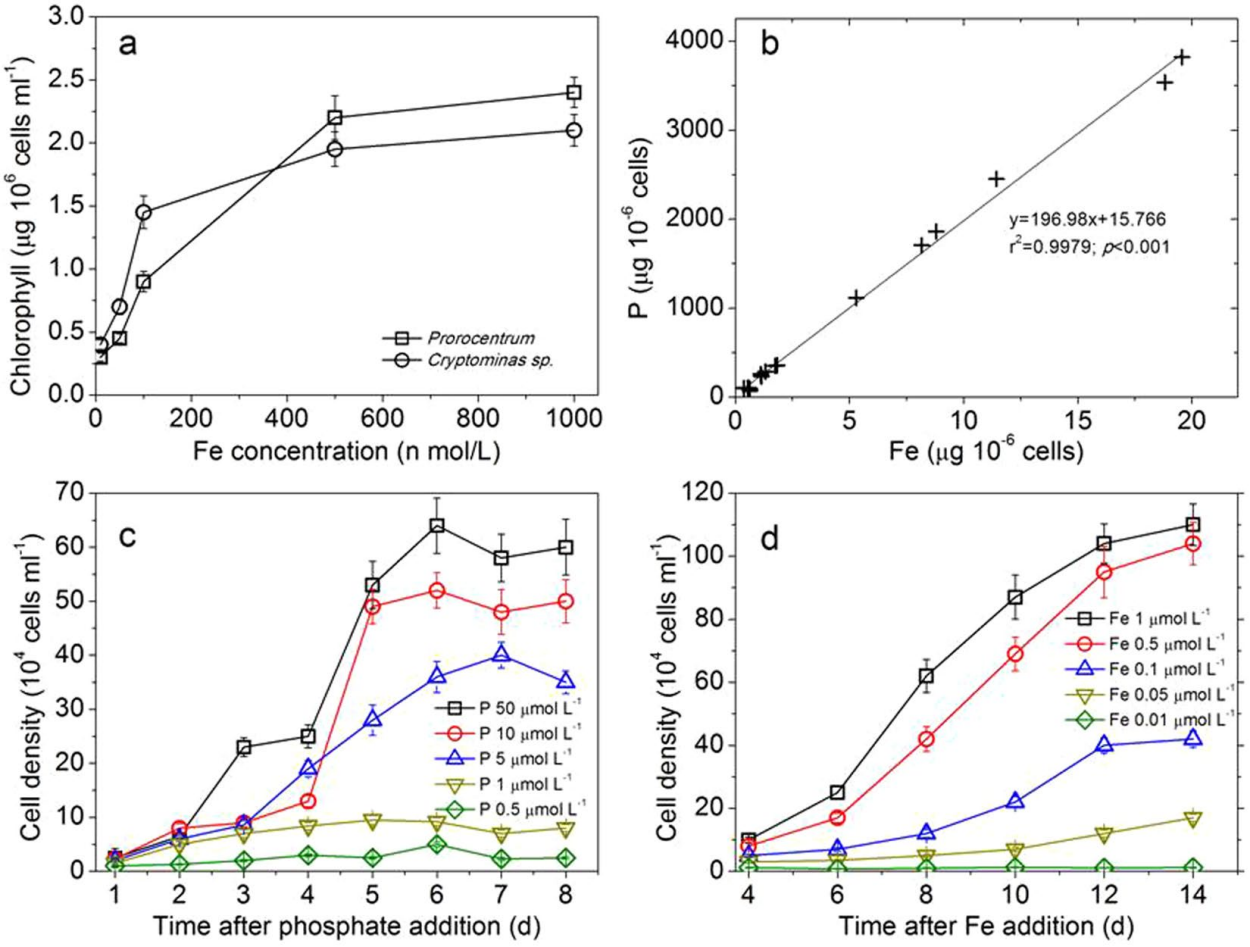
Rate-limiting effect of iron and phosphate on algae growth. (**a**) chlorophyll *a* in algae incubation as a function of Fe concentration; (**b**) phosphate and iron relationship in the cells of *Cryptomonas sp*. (**c**) cell density variations under with different P concentrations (Fe concentration of 1 μmol L^−1^); (**d**) cell density variations under different Fe concentrations (P concentration of 10 μmol L^−1^).

Fe and P are two essential nutrients for phytoplankton metabolism, which are required for photosynthetic carbon acquisition and the nitrate reductase synthesis^34,35^. *Cryptomonas* sp and *Prorocentrum micans* Ehrenberg are important algae species in the formation of HABs in the East China Sea. Results showed that when Fe < 0.1 μmol L^−1^, the measured chlorophyll concentration in incubated *Cryptomonas* sp and *Prorocentrum micans* Ehrenberg was relatively low, suggesting that algal growth rate was probably limited^36^. As a comparison, it increased significantly when Fe > 0.5 μmol L^−1^ (Fig. 2a), which is consistent with previous observations^37,38^. Under rich iron condition (1 μmol L^−1^) but relatively low P concentration (0.5–1.0 μmol L^−1^), the cell growth was significantly inhibited. Similarly, under condition of rich phosphorus (10 μmol L^−1^) but relatively low iron (0.01–0.1 μmol L^−1^), the algal growth (Fig. 2c) was slow, and the maximum cell number was much lower than the case without Fe and P limitations. The results indicated that explosive algal growth will probably be limited if Fe and P in the East China Sea cannot be supplied continuously. When Fe (1 μmol L^−1^) and P (5–50 μmol L^−1^) were both not limiting, phytoplankton cells multiplied exponentially after three days of incubation. Also, the P and Fe concentrations of the phytoplankton cells show an excellent correlation (r = 0.9979, p < 0.001) (Fig. 2d) with a molar ratio of 356:1 for P to Fe, indicating that both P and Fe are needed for cell construction and metabolic processes of algae^35^. It should be noted that our experimental conditions were not exactly the same as those in real oceanic environments. Consequently the limiting nutrient concentrations we showed here may be different from the limiting threshold for algae growth in ocean. What can be inferred from the experiments is that the high nutrient concentrations benefit the algae growth, and decreasing nutrient concentration will probably decrease algae growth.

### Light condition for algal blooms

**Figure 3:**
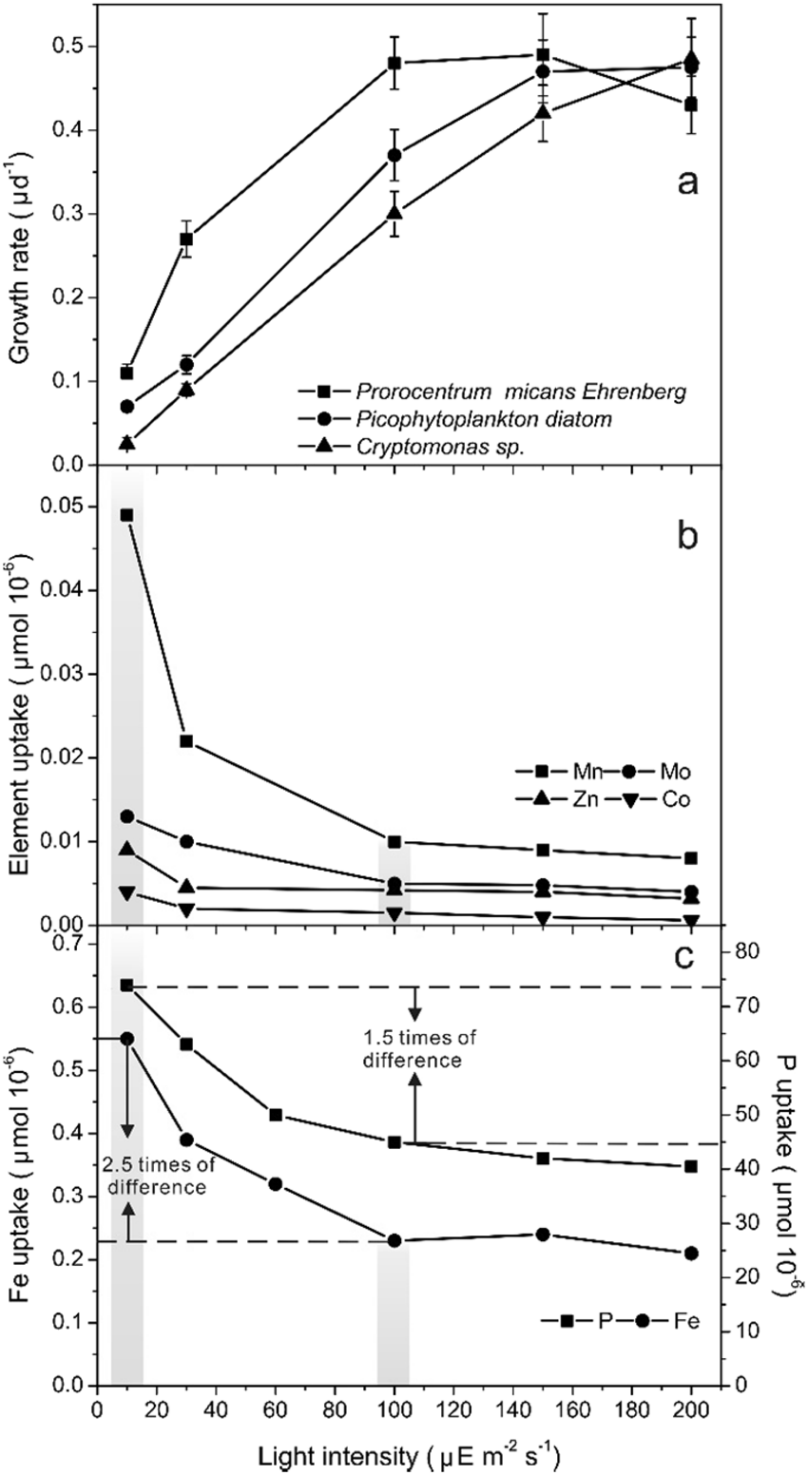
(**a**) Algae growth rate as a function of light intensity; (**b**) microelements assimilation rates as a function of light intensity; (**c**) Fe and P assimilation rates as a function of light intensity.

Light intensity is another important condition for algal bloom, especially for the turbid coastal water in the East China Sea^39,40^. The downward air flow not only makes air-borne nutrients available, but will also lead to a clear weather condition^41^, and will probably increase euphotic zone depth in turbid coastal waters. In our experiment, the specific growth rates of *Prorocentrum micans* Ehrenberg, *Cryptomonas* sp., and diatom cells increased with increasing light intensity until reaching to a plateau (saturation) (Fig. 3a). The saturation light intensity was different for different algal species. An ideal saturation light intensity of 200 μE m^−2^ s^−1^ was observed for *Prorocentrum micans* Ehrenberg, and 150 μE m^−2^ s^−1^ for *Cryptomonas* sp. and diatom cells.

Figure 3b,c showed the effect of light intensity on the uptakes of Co, Mn, Mo, Zn, and Fe, P by *Cryptomonas* sp, respectively. Increase uptakes of Fe, P, and microelements were observed under lower light intensity than under saturated light. For example, under light intensity of 10 μE m^−2^ s^−1^, the uptakes of Fe, P, Zn, Mn, Co and Mo were 2.5, 1.9, 1.8, 2.0, 2.8 and 3.5 times as large as those under 100 μE m^−2^ s^−1^, respectively. Under low-light conditions, algae cells absorb more Fe, P, Zn, Mn, Co and Mo than under strong illumination. The results were consistent with the findings that under low light, algae will improve the light absorption efficiency by increasing the surface area of the thylakoid membranes and the number of pigment protein complex^42,43^. Consequently, lots of Fe and P were stored in individual cells. On the other hand, under intense light condition, assimilated Fe, P and essential trace elements were used for organism growth and split, which subsequently decreased Fe and P content per cell. Our results indicated that the algae growth was less limited by Fe and P availability under stronger light condition and explained why HABs always occurred on sunny days.

## Discussion

**Figure 4:**
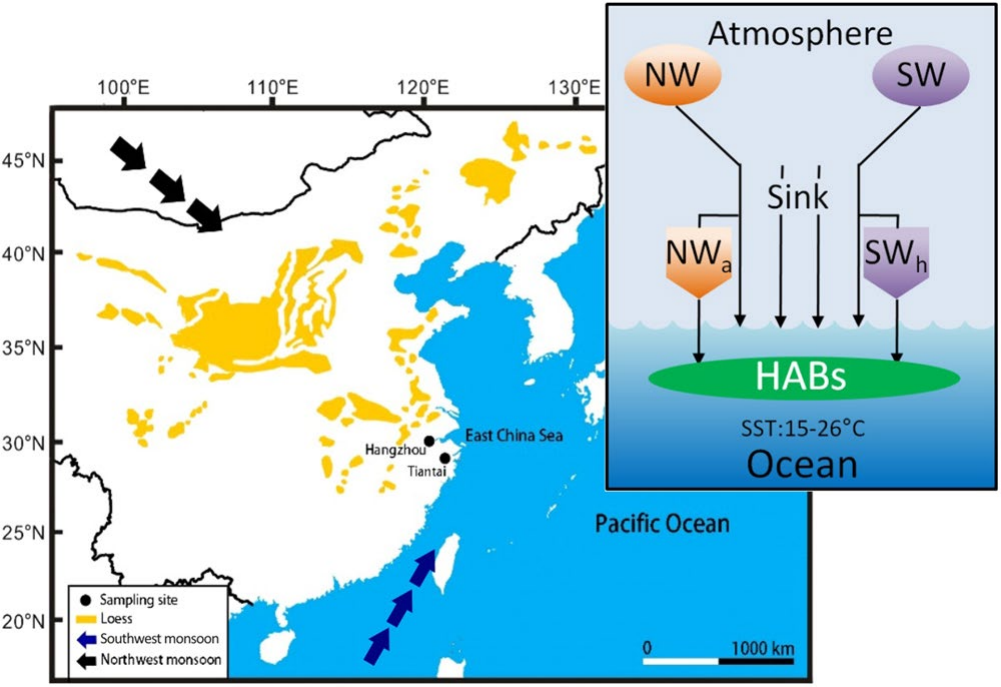
Climate stimulation model of HABs. NW: northwest monsoon; SW_h_: southwest monsoon; Sink: downward air; NW_a_: particles and nutrients carried by northwest monsoon; SW_h_: warm temperature and moist air carried by southwest monsoon; SST: surface seawater temperature. Field observation stations for collecting atmospheric particles are on the road of aerosol migration from inland China to East China Sea in the HABs months. The arrows indicate the air movement directions during the HABs events. The map was created using Surfer software v.12 Surfer, Golden Software (http://www.goldensoftware.com/products/surfer).

Most large-scale HABs occurred from April to September (Fig. 1a) when surface seawater has ideal temperature range for the growth of algae in the East China Sea (15–26 °C), and when the direction of monsoons is in a transition period between northwest and southwest in the East China Sea. The northwest monsoon transports nutrients-rich aerosol from northwestern China, especially from the Loess Plateau where particles contain an average of 4.17% of Fe_2_O_3_ and 0.95% of FeO^13^. During the transition period, the warm southwest monsoon carried moist and warm air from southwest, and generated ideal sea temperature conditions, whereas northwest monsoon transported nutrients-rich aerosol from northwest for algae growth. The contribution of various factors to the formation of HABs was summarized in Fig. 4 and can be described by the following equation:

$${\rm{HABs}}={{\rm{NW}}}_{{\rm{a}}}+{{\rm{SW}}}_{{\rm{h}}}+{\rm{SST}}+{\rm{Sink}}$$

where NW_a_ denotes particles and nutrients carried by northwest monsoon, SW_h_ denotes warm and moist air carried by southwest monsoon, SST denotes surface seawater temperature, and Sink denotes downward air. In this model NW_a_, SW_h_, SST, Sink can be obtained from meteorological observations, enabling the weather-like forecast of HABs events. Modern monitoring technologies can predict and observe the spatial and temporal distribution of aerosol events, and vertical variation of air flow dynamics. Based on these monitoring data, the described interaction of weather condition and nutrients transport on algal growth, the occurrence place, time and scale of HABs can probably be forecasted. Thus such weather-like forecast of HABs events can be used to minimize the finical loss caused by HABs.

## Materials and Methods

### Time series data collection

HABs records in the East China Sea from 2005 to 2013 were collected from The Bulletin of China Marine Environmental Status^19^ and The Communiqué on China’s Marine Disasters^44^. The concurrent meteorological data were collected from the National Centers for Environmental Prediction (NCEP), National Oceanic and Atmospheric Administration (NASA, http://www.cdc.noaa.gov). The meteorological data were daily averaged and re-analyzed. The aerosol index (AI) data were collected from the data set based on Ozone Monitoring Instrument (OMI), which was operated by the Goddard Space Flight Center, NASA (ftp://jwocky.gsfc.nasa.gov/).

### Collection and analysis of atmospheric particles

Two field observation stations were established for collecting atmospheric particles in Hangzhou and Tiantai cities. Both of them are far away from urban atmospheric pollution sources (Fig. 4). The sampling duration was from January to December 2005 in Hangzhou and from April 2005 to April 2006 in Tiantai. The atmospheric particles were collected by filtration using 0.45-µm glass fiber membrane. For each sample collection, air was continuously inhaled into a large-flow air sampler from 8:00 am. to the next day 8:00 am. at a constant flow rate of 1.0 m^3^ min^−1^.

The concentrations of Fe, P, Zn, Cu, Co, Mn, and Cr in the flask solution were determined by inductively coupled plasma–mass spectrometry (ICP–MS) (Fig. S2). Firstly, a square piece (4 × 4 cm) was cut off from a particle-bearing membrane and then was placed in a 25 ml Teflon beaker. Subsequently, a 6 ml HNO_3_–HClO_4_ mixture with a ratio of 4:2 (v/v) was added to the beaker, which was covered with a glass dish. The acid-treated and dissolved membrane were heated at 170 °C on a temperature-controlled electrothermal plate for about 5 h and then cooled to room temperature. After cooling, the membrane, beaker and the glass dish were rinsed three times with deionized water. The mixture solution and rinsed water were transferred into a 50 ml volumetric flask and diluted to 50 ml with deionized water for analysis.

Selected atmospheric particles were also examined by transmission electron microscope (TEM) to determine particle morphology and aggregation. In addition, the chemical compositions of whole particles on filters were characterized using electron microprober (EMF). A JEOL JXA-8530F Field Emission HyperProbe Electron Probe Microanalyzer equipped with an energy-dispersive X-ray spectrometer, operating in backscattered electron emission mode at 20 keV was used in this study. At least three randomly selected areas on each sample were measured. Conventional standard ZAF (atomic number, mass absorption and fluorescence) correction was carried out automatically for semi-quantitative energy dispersive spectroscopy (EDS) analysis.

### Laboratory algal incubation

Independent experiments were performed to determine the growth of HABs-related species *Cryptomonas* sp., *Prorocentrum micans* Ehrenberg, and diatom, under different iron and phosphorus concentrations. Algae *Prorocentrum micans* and *Cryptomonas* sp. were incubated in 500 mL media in 1000 mL glass bottles, while diatom was incubated in polyethylene bottles to avoid the effect of glass on algae growth. The growth media was the modified artificial sea water with chemical compositions provided in Table S1^45^. The growth experiments were performed in a light incubator with light intensity of 60 μE m^−2^ s^−1^ and light(L)/dark(D) time ratio of 12/12 (hours) at temperature of 21 ± 0.5 °C, pH of 8.0, and salinity of 30.

The effects of light intensity on the algal growth were also evaluated. Daily specific growth rate *µ* was calculated using the following formula^46^: *μ* = (ln*N*_*t*2_ − ln*N*_*t*1_)/Δ*t*, where *N*_*t*1_ and *N*_*t*2_ are cell numbers at two different times during experiment and Δ*t* is the time interval (in days) between *N*_*t*1_ and *N*_*t*2_. The uptake of iron, phosphorus, and micronutrients by *Cryptomonas* sp. were also investigated under different light intensity.

## Electronic supplementary material

Supplementary Information
