# Supplementary material for: Algae explosive growth mechanism enabling weather-like forecast of harmful algal blooms
Source: Sci Rep. 2018 Jul 2;8:9923. doi: 10.1038/s41598-018-28104-7 (PMC6028439; doi:10.1038/s41598-018-28104-7)
Supplement: Supplementary file 1 — Supplementary Information [file 41598_2018_28104_MOESM1_ESM.docx]

# Algae explosive growth mechanism enabling weather-like forecast of harmful algal blooms

Rongxiang Tian^1^, Jianfang Chen^2^, Xiangwei Sun^1^, Dewang Li^1,2^, Chongxuan Liu^3*^, Huanxin Weng^1*^

**Affiliations:**

*1 Institute of Environment & Biogeochemistry, Zhejiang University, 310027 Hangzhou, China*

*2 Key Laboratory of Marine Ecosystem and Biogeochemistry, Second Institute of Oceanography, SOA. Hangzhou 310012, China*

*3 School of Environmental Science and Engineering, Southern University of Science and Technology, Shenzhen, 518055, China*

* Corresponding author: Huanxin Weng ([gswenghx@zju.edu.cn](mailto:gswenghx@zju.edu.cn)) and Chongxuan Liu ([liucx@sustc.edu.cn](mailto:liucx@sustc.edu.cn))

## Supplementary Information

The chemical compositions of artificial sea water are shown in Table S1[^45^](#_ENREF_45). The collection and analysis procedures of surface nutrient samples in the East China Sea can be seen in Wang et al.[^16^](#_ENREF_16).

Fig. S3 showed the profiles of aerosol distribution and vertical air flow velocity (ω) during two HABs events. On June 3 to 5, 2005, a HABs event (2000 km^2^) occurred around Taohua island, Xiachi island and Jiu island of East China sea. Another HABs event (2100 km^2^) occurred on June 12 to 14, 2006 around Dongtou island and Beilu islands, East China Sea. Explosive growth of *Prorocendrum dentatum stein and Karcnia mikimotoi* caused both HABs events. Aerosols and downdraft air flow occurred before the HABs events.

**Table S1 Modified artificial sea water growth media**[**^45^**](#_ENREF_45)

| **Ingredient** | **Concentration (μmol L^-1^)** | **Ingredient** | **Concentration (μmol L^-1^)** |
| --- | --- | --- | --- |
| NaCl | 363000 | ZnSO_4_.7H_2_O | 0.254 |
| Na_2_SO_4_ | 25000 | CoSO_4_.7H_2_O | 0.00569 |
| KCl | 8040 | MnSO_4_.4H_2_O | 2.420 |
| NaHCO_3_ | 2070 | Na_2_MoO_4_.2H_2_O | 0.00610 |
| KBr | 725 | Na_2_SeO_3_ | 0.00100 |
| H_3_BO_3_ | 372 | NiCl_2_.6H_2_O | 0.00630 |
| NaF | 65.7 | CuSO_4_.5H_2_O | 0.0400 |
| MgCl_2_.6H_2_O | 41200 | Na_2_EDTA.2H_2_O | 8.29 |
| CaCl_2_.2H_2_O | 9140 | Thiamine-HCl | 0.297 |
| SrCl_2_.6H_2_O | 82 | Biotin | 0.00409 |
| NaNO_3_ | 549000 | V_B12_ | 0.00147 |
| NaH_2_PO_4_.H_2_O | 21000 |  | |

**Figure legends**

**Figure S1.** **Distributions of phosphate (a), nitrate (b), and silicate (c) concentrations in surface East China Sea.** The black dots in Fig. S1 denoted sampling locations. The red circles showed the positions of past HABs in the East China Sea, according to Zhou et al[^31^](#_ENREF_31). The circle sizes represented roughly the relative spatial scale of HABs. The figure was created using Ocean data view 4.7.4 (http://odv.awi.de/).

**Figure S2. Atmospheric total suspended particle (TSP) and microelement concentrations during HABs in 2005**

**Figure S3. Spatial distributions of aerosols (a, b) and vertical air flow velocity (ω) (c and d) during two HABs events.** Figs S3a and 3c showed HABs on June 2, 2005, 122.5E, Figs S3b and 3d showed HABs on June 6, 2006, 122.15E. Vertical dash lines in Figs S3a and 3b denote the longitude values of air flow transect in Figs. S3c and 3d, respectively. In Figs S3c and 3d, if the vertical velocity of airflow ($\omega=\frac{dP}{dt}$) is larger zero (i.e., ω>0), it is downdraft, otherwise, it is updraft. The figure was created by GrADS version 1.8 (Grid Analysis and Display System, http://cola.gmu.edu/grads)**.**
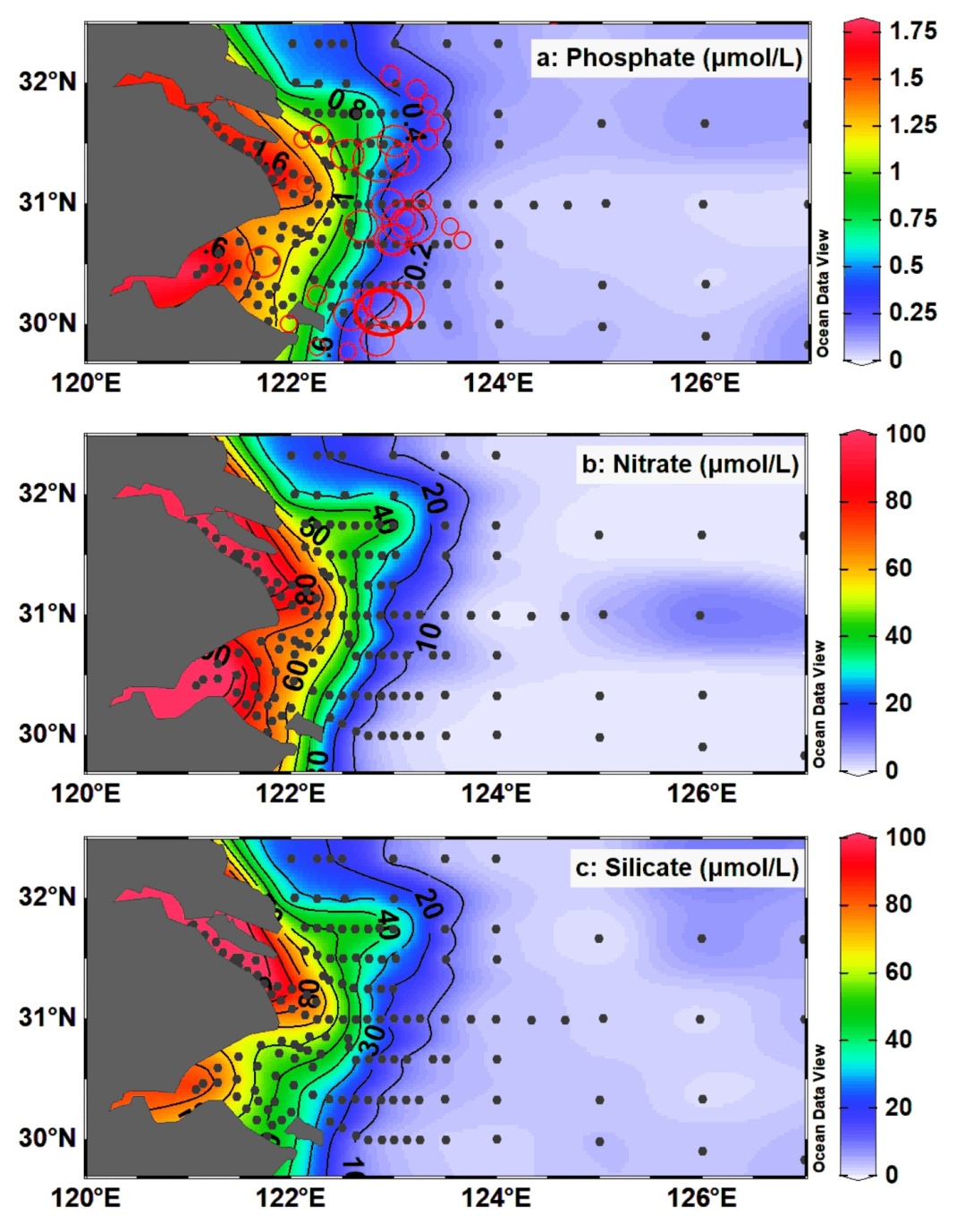


**Figure S1.** **Distributions of phosphate (a), nitrate (b), and silicate (c) concentrations in surface East China Sea.** The black dots in Fig. S1 denoted sampling locations. The red circles showed the positions of past HABs in the East China Sea, according to Zhou et al[^31^](#_ENREF_31). The circle sizes represented roughly the relative spatial scale of HABs. The figure was created using Ocean data view 4.7.4 (http://odv.awi.de/).

**
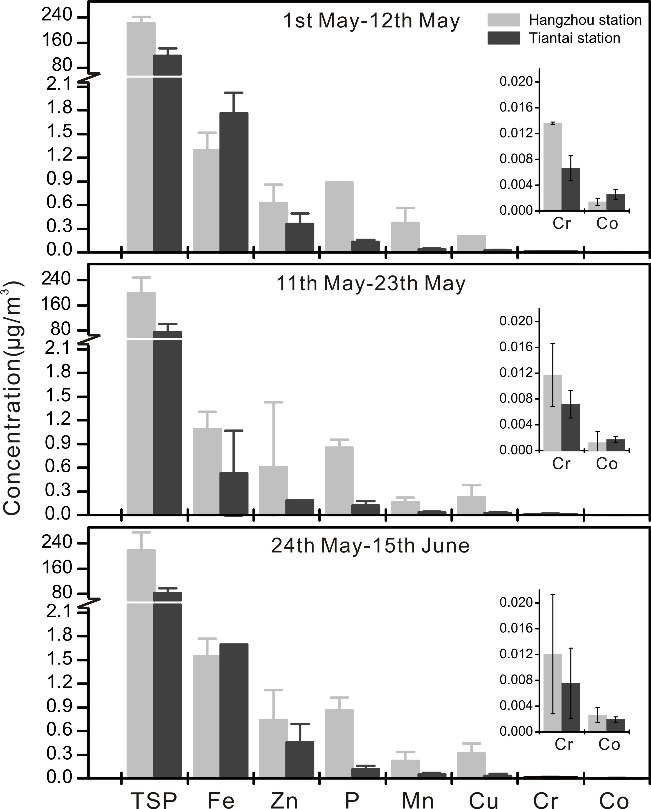
**

**Figure S2. Atmospheric total suspended particle (TSP) and microelement concentrations during HABs in 2005.**


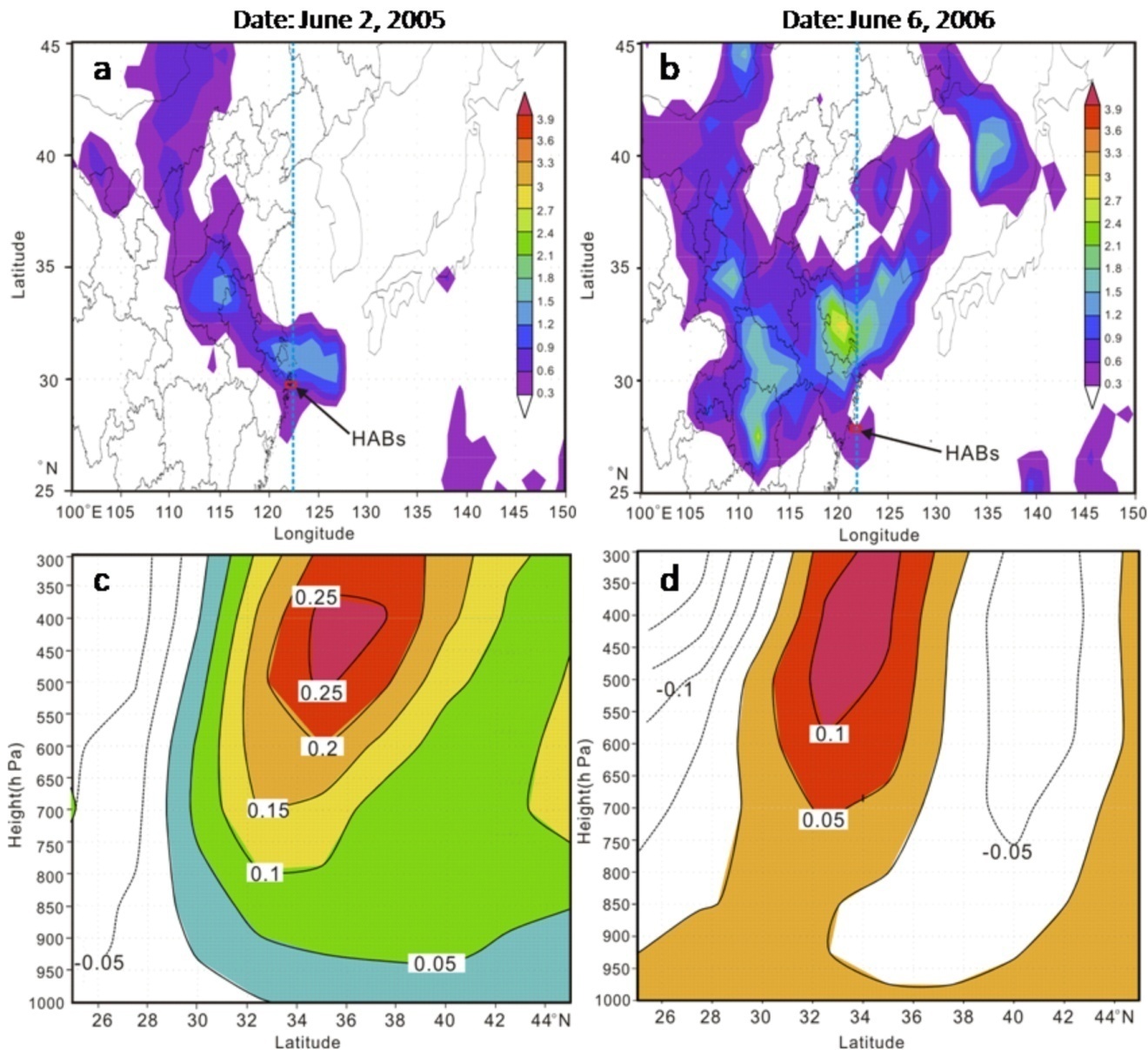


**Figure S3. Spatial distributions of aerosols (a, b) and vertical air flow velocity (ω) (c and d) during two HABs events.** Figs S3a and 3c showed HABs on June 2, 2005, 122.5E, Figs S3b and 3d showed HABs on June 6, 2006, 122.15E. Vertical dash lines in Figs S3a and 3b denote the longitude values of air flow transect in Figs. S3c and 3d, respectively. In Figs S3c and 3d, if the vertical velocity of airflow ($\omega=\frac{dP}{dt}$) is larger zero (i.e., ω>0), it is downdraft, otherwise, it is updraft. The figure was created by GrADS version 1.8 (Grid Analysis and Display System, http://cola.gmu.edu/grads)**.**
